# A Novel Gene Coding γ-Aminobutyric Acid Transporter May Improve the Tolerance of *Populus euphratica* to Adverse Environments

**DOI:** 10.3389/fpls.2019.01083

**Published:** 2019-09-11

**Authors:** Xiaotao Bai, Jianmei Xu, Xuemin Shao, Wenchun Luo, Zhimin Niu, Chengyu Gao, Dongshi Wan

**Affiliations:** State Key Laboratory of Grassland Agro-Ecosystem, School of Life Sciences, Lanzhou University, Lanzhou, China

**Keywords:** γ-aminobutyric acid transporters, novel gene, functional divergence, poplars, adaptive evolution

## Abstract

Novel genes provide important genetic resource for organism innovation. However, the evidence from genetic experiment is limited. In plants, γ-aminobutyric acid (GABA) transporters (GATs) primarily transport GABA and further involve in plant growth, development, and response to various stresses. In this study, we have identified the *GATs* family in *Populus* species and characterized their functional evolution and divergence in a desert poplar species (*Populus euphratica*). We found that the *GATs* underwent genus-specific expansion *via* multiple whole-genome duplications in *Populus* species. The purifying selection were identified across those *GATs* evolution and divergence in poplar diversity, except two paralogous *PeuGAT2* and *PeuGAT3* from *P. euphratica*. The both genes arose from a tandem duplication event about 49 million years ago and have experienced strong positive selection, suggesting that the divergence in PeuGAT3 protein function/structure might define gene function better than in expression pattern. Both *PeuGAT* genes were functionally characterized in *Arabidopsis* and poplar, respectively. The overexpression of *PeuGAT3* increased the thickness of xylem cells walls in both *Arabidopsis* and poplar and enhanced the lignin content of xylem tissues and the proline accumulation in poplar leaves, all of which may improve tolerance of salt/drought stress in desert poplars. Our findings help clarify the genetic mechanisms underpinning high tolerance in desert poplars and suggest that *PeuGAT3* could be an attractive candidate gene for engineering trees with improved brown-rot resistance.

## Introduction

Novel genes provide important genetic resource for organism innovation. The novel genes may originate from various models, such as gene duplication, exon shuffling, and retroposition, which all may play important roles in plant evolution (Long et al., 1999). The novel gene often evolves rapidly and diversify in function, including expression pattern, location after translation, and biochemical function (Tautz and Domazet-Lošo, 2011), which all contribute to the formation of their specific traits in species or lineage, such as tolerance to stresses (Force et al., 1999; Shukla et al., 2014). The divergence of the novel genes in function may occur in multiple levels (Force et al., 1999; Hittinger and Carroll, 2007; Gagnon-Arsenault et al., 2013; Nan et al., 2017). In general, the primary mechanisms responsible for functional divergence include site-specific regulatory modification (Bergström et al., 2014) and changes in enzymatic activity and protein specificity (Force et al., 1999; Voordeckers et al., 2012). Of them, motif loss could potentially lead to changes in protein structure, which could in turn alter the function of protein.

Amino acid transporters (AATs) are membrane-integrated proteins that mediate the transport of amino acids across cellular membranes in flowering plants and play indispensable roles in various processes of plant growth and development (Wipf et al., 2002; Tegeder, 2012). There are two major families of plant AATs: the amino acid/auxin permease (AAAP) family and the amino acid–polyamine–choline transporter family (APC) (Ortiz-Lopez et al., 2000; Saier et al., 2008). The AAAP family has at least six subfamilies, including amino acid permeases (AAPs), lysine and histidine transporters (LHTs), proline transporters (ProTs), γ-aminobutyric acid transporters (GATs), auxin transporters (AUXs), and aromatic and neutral amino acid transporters (ANTs) (Okumoto and Pilot, 2011). Those subfamilies, refer to *Arabidopsis*, define with the type of the transporting amino acids/or metabolite molecular and diversify in the number of motifs of coding region and spatial–temporal expression, thus involve widely in various processes of plant growth and development (Schwacke et al., 1999). In AAAP family, GATs mainly transport the ubiquitous amino acid γ-aminobutyric acid (GABA) (Narayan and Nair, 1990), which act as a signal molecule that affects C/N balance, pH regulation, nitrogen storage, and defense mechanisms (Shelp et al., 1999; Bouche and Fromm, 2004). For example, GABA accumulation can modulate the activity of plant-specific anion transporters, cause changes in root growth, and improved salt stress tolerance (Bouche and Fromm, 2004). Only one GAT1 transporter has been identified in *Arabidopsis*; it cannot transport proline but has a high affinity for GABA (Schwacke et al., 1999), and are suggested to mediate interactions between carbon and nitrogen (Batushansky et al., 2015). Interestingly, in *Populus*, more putative *GAT* copies were identified in the genome of black cottonwood (Tuskan et al., 2006), *Populus euphratica* (Ma et al., 2013) and *Populus alba* (Ma et al., 2019). It seems clear that a genus-specific expansion of GAT genes occurred at some point in the evolutionary history of the poplars and that the duplicated *GATs* diversified, thereby avoiding functional redundancy and leading to behaviors that improve the salt tolerance of woody plants and increase their xylem tissue growth.

*P. euphratica* Oliv. is mainly distributed in central Asia and prefers dry and central deserts with deep underground water sources (Ottow et al., 2005). As an important tree with high resistance to salt and drought, *P. euphratica* has been studied extensively in efforts to understand stress resistance mechanisms in woody plants. Its leaves are phenotypically polymorphic: they become succulent under salt stress, which increases their ability to tolerate highly saline conditions (Ottow et al., 2005). *P. euphratica* also has a well-developed root system with a high capacity for hydrotropism, increasing its access to ground water and its likelihood of surviving in desert ecosystems. Finally, its wood is rigid and dense, helping to prevent excessive evaporation of water. The publication of genomic data for *P. euphratica* led to the identification of several gene families associated with responses to various stresses that have expanded *via* gene duplication events followed by functional diversification, such as HKT (Ma et al., 2013), and WRKY (Ma et al., 2016) families. These studies show that many of the genes responsible for the high stress resistance of *P. euphratica* arose *via* gene duplication and subsequent functional divergence, which may confer an adaptive advantage for desert poplars in arid and saline environments (Perry et al., 2007). Therefore, more research is needed to identify genetic resources that could be exploited in molecular tree breeding.

Here, we present a phylogenetic analysis of a set of GAT-like proteins in *Populus*. By inferring from phylogeny trees, we show that the GAT genes probably underwent specific-genus expansion in *Populus* species. To better characterize the function among GATs in poplar, a novel gene arose by gene duplication, and its paralogs from *P. euphratica* have been overexpressed in *P. alba* var. *pyramidalis* and *Arabidopsis thaliana* (col-0), respectively. We then compared the effect on plant growth, plant wood properties, wood structure, and proline accumulation under salt stress. Our results provide highlight insight into the role of GAT novel gene in the response to environment stress *P. euphratica*. 

## Materials and Methods

### Plant Materials and Salt Treatments

Two-year-old seedlings of *P. euphratica* were obtained by germinating seeds collected from Akesu in Xinjiang province, China. Two-year-old saplings of *P. alba var. pyramidalis* were obtained from cuttings. All seedlings and saplings were cultivated in pots with loam soil and grown under greenhouse conditions with a constant temperature of 25°C, a 16-h (16/8 h) photoperiod with a photon flux of 80 µmol m^−2^ s^−1^ and 60% relative air humidity. Saplings and seedlings from two poplars with similar growth statuses were treated with 0, 150, and 300 mM NaCl solutions for a week under the same growth conditions (150 and 300 mM, representing moderate and severe stresses, respectively). All samples were collected and examined to determine the treatments’ physiological effects.

### Phylogenetic Analyses of GAT Genes in Plants

*GAT* genes were identified by TBLASTN searches of the five genome databases *P. euphratica* (Ma et al., 2013), *P. alba* (Ma et al., 2018), *P. trichocarpa* (Tuskan et al., 2006), *P. pruinosa* (Ma et al., 2016), and willow (Dai et al., 2014) using 22 full-length AAAP sequences from *Arabidopsis* (*Arabidopsis thaliana*; Dixon and Edwards, 2010a) with an e-value cut-off 1-E-30. These candidate full-length AAAPs were primarily analyzed using the NCBI (https://www.ncbi.nlm.nih.gov/Structure/cdd/wrpsb.cgi) and MEME (http://meme-suite.org/doc/overview.html) conserved domain search to identify typical AAAP Aa_trans domain in their protein structures. When an AAAP candidate was identified to contain typical AAAP Aa_trans domain in its protein structure, it was considered as an AAAP gene. A total of 188 AAAPs (**Table S3**) represent six clades (GAT, ProT, LHT, AAP, AUX, and ANT) defined by the NCBI conserved domain database (Marchler et al., 2005) and MEME methods (Bailey et al., 2006). Subsequently, these Aa_trans domain sequences of candidate genes were aligned using CLUSTALW (Tamura et al., 2007). Phylogenetic analyses were conducted using the neighbor-joining (NJ) method as implemented in the MEGA version 4.1 software package, using the pairwise deletion option to handle alignment gaps and the Poisson correction model for distance computation. Bootstrap tests were performed using 1,000 replicates. The branch lengths in the resulting phylogenetic tree are proportional to phylogenetic distances. The rate of nonsynonymous substitution rate (Ka) and synonymous substitution (Ks) of paralogs were calculated using DNAsp version 6 (Rozas et al., 2017). Transmembrane domain analysis was performed using the TMHMM server version 2.0 (http://www.cbs.dtu.dk/services/TMHMM/), and protein tertiary structure prediction was performed using the SWISS-MODEL (https://swissmodel.expasy.org/).

#### PeuGAT2 and PeuGAT3 Overexpress in Arabidopsis and Poplars

*PeuGAT2* (CCG029175.1) and *PeuGAT3* (CCG029176.1) coding regions were amplified using specific primers (**Table S1**), subcloned into the PENTR/D TOPO vector (Invitrogen, Carlsbad, CA, USA), and then transferred into the GATAWAY destination pk7WG2D.1 vector (Karimi et al., 2002). In the latter vector, the CaMV 35S promoter drove the expression of the gene of interest (*PeuGAT2* and *PeuGAT3*, respectively) and the reporter gene green fluorescent protein (GFP) in an LR Clonase reaction (Invitrogen, Carlsbad, CA, USA). The 35S::*PeuGAT2* and 35S::*PeuGAT3* constructs were introduced into the *Agrobacterium tumefaciens* strain GV3101 and then transformed into wild-type (WT) *Populus* species (*P. alba*) using the leaf disk method (Horsch et al., 1985) with some modifications (Ma et al., 2019). Putative transgenic plants were selected on Murashige and Skoog (MS) agar (0.8%, w/v) medium containing 100 mg/l kanamycin, and successful transformation was verified by semiquantitative reverse transcription PCR (RT-PCR) using *PeuGAT2*- and *PeuGAT3*-specific primers. The transcript levels were normalized to endogenous ACTIN transcripts amplified with primers Actin F/R (**Table S1**). Each set of experiments was repeated three times, and the relative quantification method (2−ΔΔCT) was used to evaluate quantitative variation (Livak and Schmittgen, 2001). We obtained eight independent transgenic lines. The putative transgenic poplar plantlets were incubated in a growth chamber at 25 ± 1°C with a 16-h-light/8-h-dark photoperiod and a relative humidity (RH) of 50–60%.

Transgenic *Arabidopsis* lines were obtained by transforming WTA. *thaliana* (ecotype Columbia-0) using floral dipping methods as described by Clough and Bent (1998). The T1 transgenic lines were screened on 1/2 MS medium containing 50 mg/L kanamycin. The selection of transgenic lines were continued until T3 generations to obtain transgenic homozygous lines with a single T-DNA locus (e.g.,*35S::PeuGAT2* and *35S::PeuGAT3*). The T-DNA insertion lines were identified using the SIGnAL T-DNA Express *Arabidopsis* Gene Mapping Tool (http://signal.salk.edu/cgi-bin/tdnaexpress), and the *PeuGAT2*- and *PeuGAT3*-specific primers (**Table S1**) were used as the screening primers. The selected transgenic seedlings were grown on MS agar (0.8%, w/v) medium for 5–7 days. A total of 16 independent transgenic *Arabidopsis* lines (nine *35S::PeuGAT2* lines and seven *35S::PeuGAT3* lines, respectively) (**Figures S2A, B**) were obtained. We found that the phenotypes of these transgenic lines were correlated with the expression levels. Therefore, *35S::PeuGAT2*L9 and *35S::PeuGAT3*L6 lines were selected for the subsequent experiments because of their high and similar expression levels. Similarly, the *35S::PeuGAT2*L9 and *35S::PeuGAT3*L4 from the confirmed transgenic poplar saplings (10*35S::PeuGAT2* and 8*35S::PeuGAT3*, respectively; **Figures S2C, D**) were selected for the subsequent experiments. The selected transgenic poplar saplings were propagated by cutting, grown for 3 weeks in culture vessels, and then transplanted to soil under a photoperiod of 16-h light/8-h dark with a relative humidity of 50–60% for 3–4 weeks at 25°C.

### Abiotic Stress Treatments of *Arabidopsis*

WT and transgenic *Arabidopsis* seeds were vernalized at 4°C for 2 days after water absorption. After disinfection with mercury bichloride solution, the seeds were planted on MS, 50mM and 100mM NaCl MS medium for germination, respectively. Five days later, the germinated seeds were separately transferred to common medium and several stress-conditioned MS medium (75 mM NaCl and 150 mM NaCl for salt treatments; 5% PEG6000 and 10% PEG6000 for drought treatments) to continue cultivate for 1 week, and then, the phenotypes of wide-type and transgenic *Arabidopsis* were determined.

### Anatomical Observations in the Transgenic *Arabidopsis* and *Populus*

For histological observations, rooted WT and transgenic plants (at least seven individuals for each line) were transplanted into soil and maintained in the greenhouse, respectively. Fresh stems from the base internode of 6-week-old *Arabidopsis* (L9 for *35S::PeuGAT2* and L6 for *35S::PeuGAT3*) and 3-month-old WT (*P. alba*) and transgenic poplars (L9 for *35S::PeuGAT2* and L4 for *35S::PeuGAT3*) (at least 2 cm in diameter) were ﬁxed with 2% formaldehyde and passed through a graded ethanol series. The stems were then embedded in paraffin and 8-µm-thick sections were cut with a rotary microtome. After removing residual paraffin, the sections were stained with 0.05% toluidine blue and examined with a light microscope. Images were captured under bright field using an ECLIPSE 80i microscope (Nikon, Tokyo, Japan). The radial widths of the phloem, cambium, and xylem were measured (at 20× and 40× magnification, respectively) using the Image Tool software package (UTHSCSA, San Antonio, TX, USA).

### The Examination of Chemical Compositions in Transgenic Poplars and Proline Level Assay

Two-year-old WT and transgenic *P. alba* saplings were grown in soil with a 16-h-light/8-h-dark cycle at 25°C and 50% relative humidity under uniform illumination. Plants with stem diameters above 2 cm were selected for further experimentation. The wood composition of the selected saplings was studied using a Bruker Tensor 27 Fourier transform infrared (FTIR) spectrometer (Bruker, Germany) as described by Rodrigues et al. (1998). Five major parameters, I1505/I1425 (the relative content of lignin), I1505/I895 (the ratio of lignin to cellulose), I1505/I1738 (the ratio of lignin to hemicellulose), and I1505/I1382 and I1505/I1158 (the ratio of lignin to total cellulose) were measured to characterize the saplings’ wood composition, while cell wall thickness was measured using a dissecting microscope to characterize their anatomical structure. Leaf materials detached from WT and transgenic *P. alba* saplings were measured to determine their Pro contents using previously reported protocols (Li et al., 2017). These experiments were independently replicated three times under identical conditions.

### Statistical Analysis

All statistical analyses were performed using the SPSS 17.0 software package. All quoted *p* values were derived using Student’s *t*-test based on one-way analysis of variance; *p* values below 0.05 and 0.01 are denoted by one asterisk (*) and two asterisks (**), respectively. Significance was evaluated by one- and two-way analyses of variance. The data were normalized, and all samples were normally distributed with homogeneity of variance.

## Results

### GATs Evolution Among *Populus* Species

**Figure 1:**
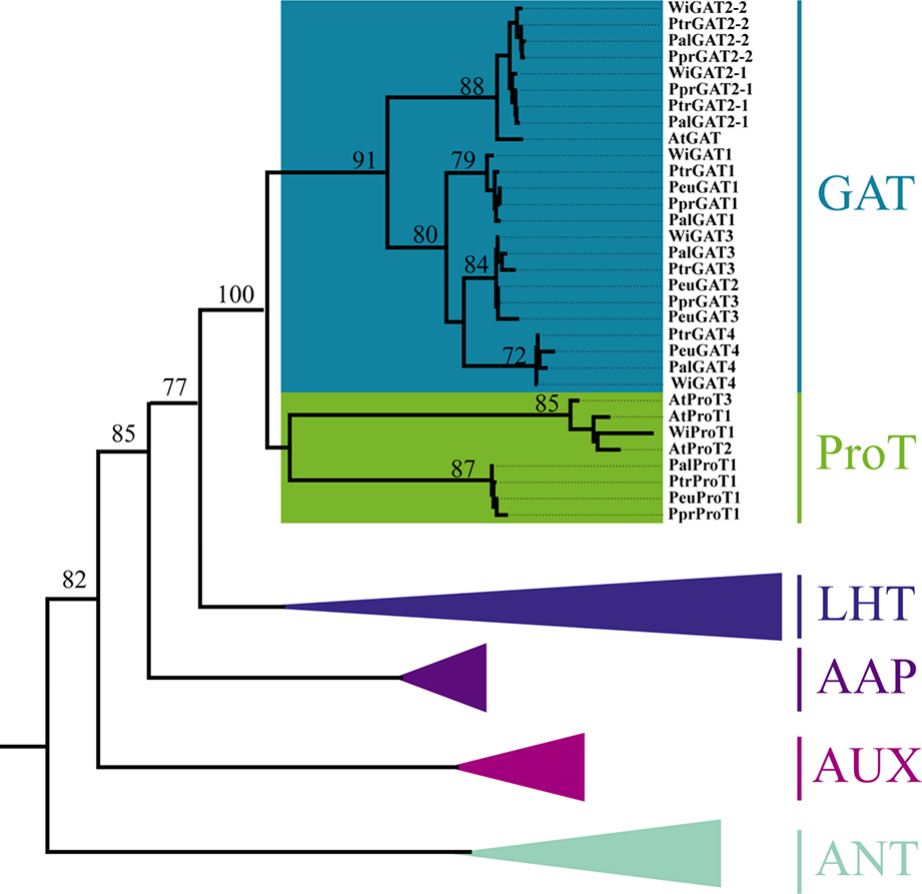
Phylogenetic position of AAAP isoforms among other transporters from *Populus* species and *Arabidopsis thaliana*. All transporter proteins were clustered into six clades, classified as AAP, LHT, AUX, ANT, ProT, and GAT transporters and marked with different background colors. Plant species are as follows: At, *Arabidopsis thaliana*; Ptr, *Populus trichocarpa*; Ppr, *P. pruinosa*; Peu, *P. euphratica*; willow, *Salix integra*.

GAT has been clustered into the AAAP family and sister with other five groups corresponding to the AAP, LHT, AUX, ANT, and ProT subfamilies (Ortiz-Lopez et al., 2000). The ProT and GAT subfamilies separated recently and clustered together. In four poplar species, a total of 18 GAT genes have been retrieved. Among them, two salt-resistive poplars (*P. euphratica* and *P. pruinosa*) have four GAT members, while five members were found in two salt-sensitive poplars (*P. trichocarpa* and *P. alba*) (**Table 1**). In *Arabidopsis*, only one GAT member has been found, whereas three ProT members have been retained (**Figure 1**). Phylogenetic analysis showed that GAT and ProT subfamilies separated from each other due to one whole-genome duplication. Then, GAT expanded *via* gene duplications with genus-specific in *Populus*. These genes have conserved protein domains similar to those found in various plant amino acid transporters, and sequence alignments revealed high homology with glutamate derived γ-aminobutyric acid transporters (GATs) from *Arabidopsis* (Batushansky et al., 2015).

**Table 1:** Ks-dating and Ka/Ks ratios of GAT paralogs from different poplar species.

Duplicated genes	Ka/Ks ratio	Duplicated genes	Ka/Ks ratio
*PeuGAT1/PeuGAT2*	0.1438	*PalGAT3/PalGAT4*	0.0106
*PeuGAT1/PeuGAT3*	1	*PtrGAT1/PtrGAT2-1*	0.0842
*PeuGAT1/PeuGAT4*	0.1463	*PtrGAT1/PtrGAT2-2*	0.3959
*PeuGAT2/PeuGAT3*	1.1747	*PtrGAT1/PtrGAT3*	0.5931
*PeuGAT2/PeuGAT4*	0.1750	*PtrGAT1/PtrGAT4*	0.5637
*PeuGAT3/PeuGAT4*	0.2023	*PtrGAT2-1/PtrGAT2-2*	0.1630
*PprGAT1/PprGAT2-1*	0.2508	*PtrGAT2-1/PtrGAT3*	0.0746
*PprGAT1/PprGAT2-2*	0.2961	*PtrGAT2-1/PtrGAT4*	0.2272
*PprGAT1/PprGAT3*	0.1367	*PtrGAT2-2/PtrGAT3*	0.2206
*PprGAT2-1/PprGAT2-2*	0.1637	*PtrGAT2-2/PtrGAT4*	0.2131
*PprGAT2-1/PprGAT3*	0.2032	*PtrGAT3/PtrGAT4*	0.4209
*PprGAT2-2/PprGAT3*	0.5273	*WiGAT1/WiGAT2-1*	0.2081
*PalGAT1/PalGAT2-1*	0.2406	*WiGAT1/WiGAT2-2*	0.2419
*PalGAT1/PalGAT2-2*	0.8139	*WiGAT1/WiGAT3*	0.1471
*PalGAT1/PalGAT3*	0.1487	*WiGAT1/WiGAT4*	0.1457
*PalGAT1/PalGAT4*	0.1333	*WiGAT2-1/WiGAT2-2*	0.1196
*PalGAT2-1/PalGAT2-2*	0.1762	*WiGAT2-1/WiGAT3*	0.8407
*PalGAT2-1/PalGAT3*	0.8042	*WiGAT2-1/WiGAT4*	0.3160
*PalGAT2-1/PalGAT4*	0.1428	*WiGAT2-2/WiGAT3*	0.8323
*PalGAT2-2/PalGAT3*	0.7459	*WiGAT2-2/WiGAT4*	0.2661
*PalGAT2-2/PalGAT4*	0.2841	*WiGAT3/WiGAT4*	0.1263

Ka, nonsynonymous substitution rate; Ks, synonymous substitution rate; Ptr, P. trichocarpa; Ppr, P. pruinosa; Peu, P. euphratica; willow, Salix integra.

### Identification of Novel Gene

**Figure 2:**
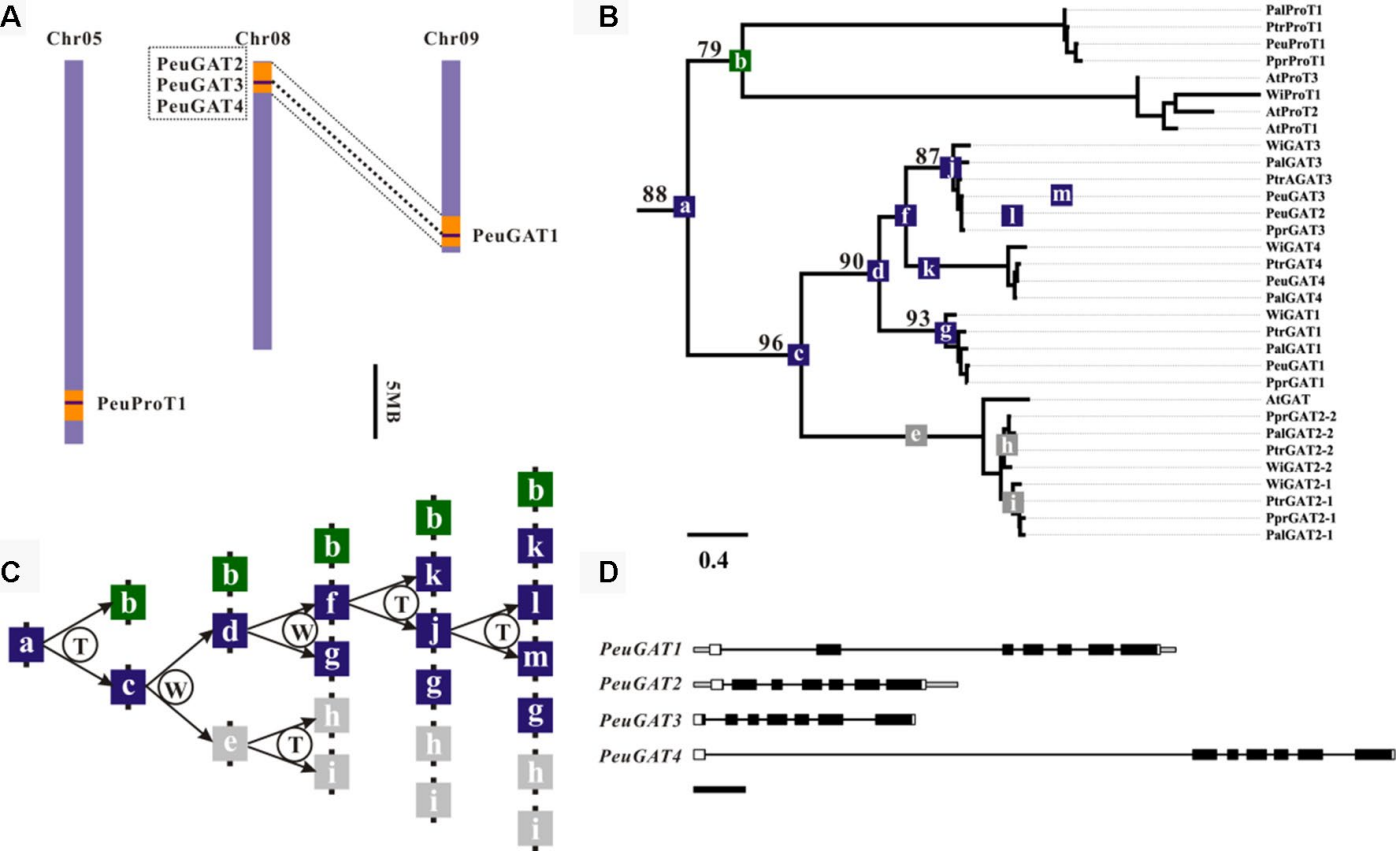
Genomic localization, phylogenetic relationships, and hypothetical duplication histories of the *PeuGAT* genes. **(A)** Regions that are assumed to correspond to homologous genome blocks are shaded in orange and connected with lines. **(B)** Phylogenetic relationships were reconstructed using the WAG + I + G model. Numbers on branches indicate the bootstrap percentage values calculated from 1,000 bootstrap replicates. **(C)** The letters T and W in the schematic diagram showing the hypothetical origins of *PeuGAT* genes indicate tandem duplication and whole-genome duplication, respectively. Gray boxes represent the presumed pseudogenes or lost genes. Blue and green boxes represent *PeuGAT* and *PeuProT* genes, respectively. **(D)** The Aa_trans-coding domain is colored black, and the remaining exon sequences are shown as white boxes. Gray boxes represent untranslated regions, while lines represent introns. Scale bar: 500 bp.

From the phylogenetic analysis as above, two *P. euphratica* genes *PeuGAT2* and *PeuGAT3* have clustered into one group, while in other species, especially for *P. pruninose*, a closely related poplar of *P. euphratica*, there was only one copy in that group; thus, *PeuGAT3* are lineage specific (**Figure 2A**). Gene colinearity analysis found that the *PeuGAT2* and *PeuGAT3* located on eighth chromosome and are separated by an interval of ∼900 bp (**Figure 2A**); this suggested that the two paralogs arose from a tandem duplication event since the recent whole-genome duplication (**Figure 2C**). Protein structure analysis showed that PeuGAT3 was partially deleted at the N-terminus compared to PeuGAT2 (**Figures S1A, B**), and this deletion resulted in the lack a transmembrane region in PeuGAT3 compared to PeuGAT2 (**Figure S1C**). The estimation of divergence times among *PeuGAT* genes using nucleotide substitution rate of 1.2 × 10^−8^ (Ingvarsson, 2008) found that *PeuGAT2* and *PeuGAT3* diversified about 49 million years ago (Mya) (**Table S2**), indicating the two genes duplicated after the recent WGD of *Populus*. Finally, the Ka/Ks ratio was used to identify positive selection for all *Populus* GAT paralogs. Most of GATs of Ka/Ks ratio were lower than 1, suggesting that the GATs suffered from strong purifying selection, while Ka/Ks ratio between PeuGAT2 and PeuGAT3 was higher than 1, indicating that both genes experienced positive selection.

### Expression Pattern of *PeuGAT2* and *PeuGAT3* in *P. Euphratica* During Salt Stress

**Figure 3:**
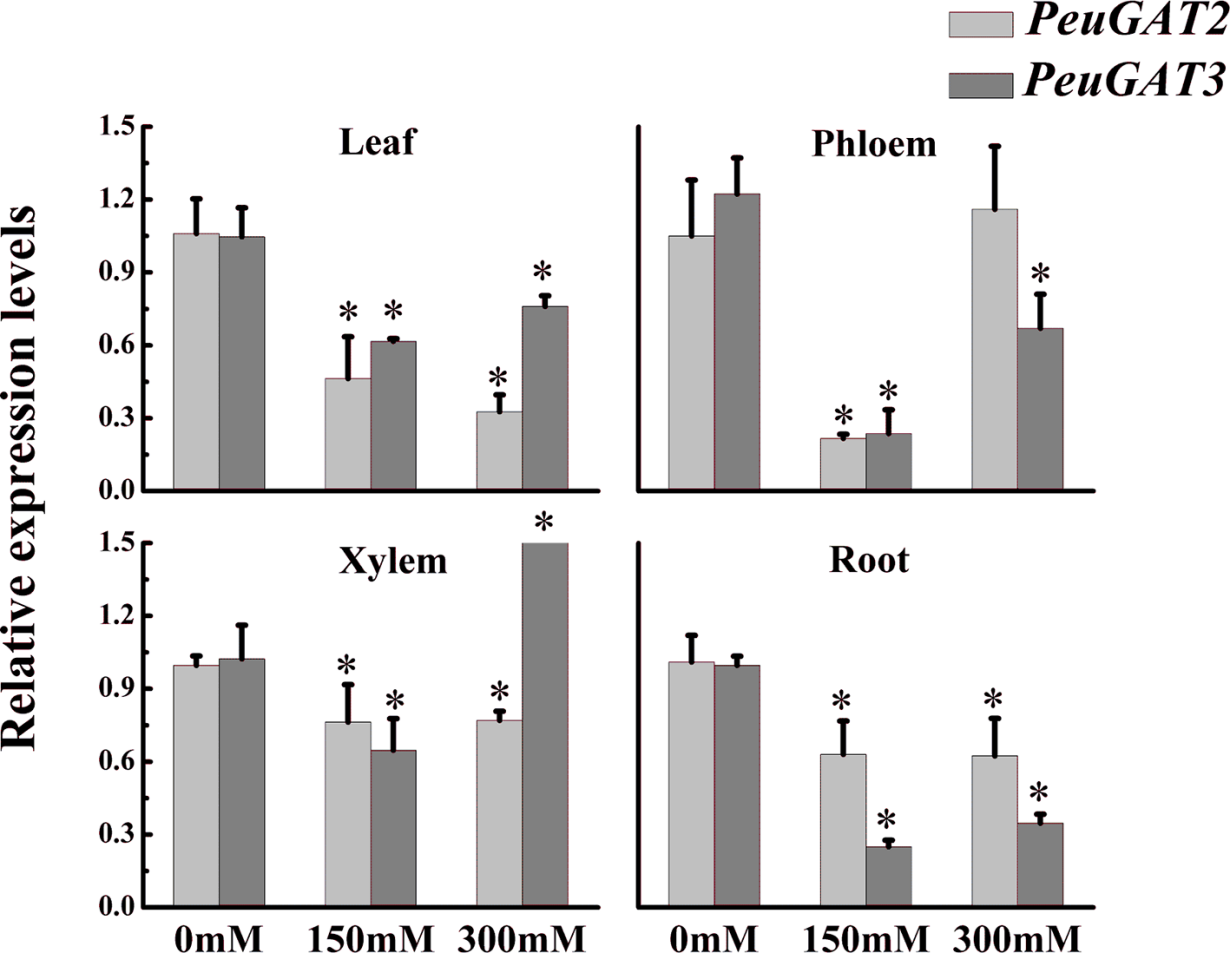
The expression pattern of *PeuGAT2* and *PeuGAT3* in *P. euphratica* seedlings treated with different concentrations of NaCl for 2 weeks. RNA was extracted from leaves, roots, phloem, and xylem, and the relative expression of the two genes was quantified by real-time PCR; 18S rRNA was used as a reference. Three replicates of each sample were analyzed. All values are means ± SE (*n* = 3). Values labeled with different letters differ significantly (*p < 0.05).

To identify potential differences in expression patterns between *PeuGAT2* and *PeuGAT3*, we quantified their expression in the stems, roots, and leaves of *P. euphratica* seedlings. Both genes were expressed simultaneously in all studied organs (**Figure 3**). However, salt stress induced by treatment with different NaCl concentrations reduced the expression of both genes in leaves and roots. Furthermore, compared with *PeuGAT3*, the overall expression of *PeuGAT2* in leaves were more severely suppressed, while in roots, it exhibited the opposite result. In xylems, the two genes exhibited similar patterns under moderate salt stress, while under severe stress, *PeuGAT3* exhibited significant higher expression level compared with *PeuGAT2*. On the contrary, *PeuGAT2* could maintain relatively high expression in phloem under severe stress. Those results show that the two genes have different expression patterns and that these differences become more pronounced in response to salt stress.

### Overexpression *PeuGAT3* Enhances Abiotic Stress Tolerance in *Arabidopsis* and Improves Lignin Accumulation in *Arabidopsis* and Poplar

**Figure 4:**
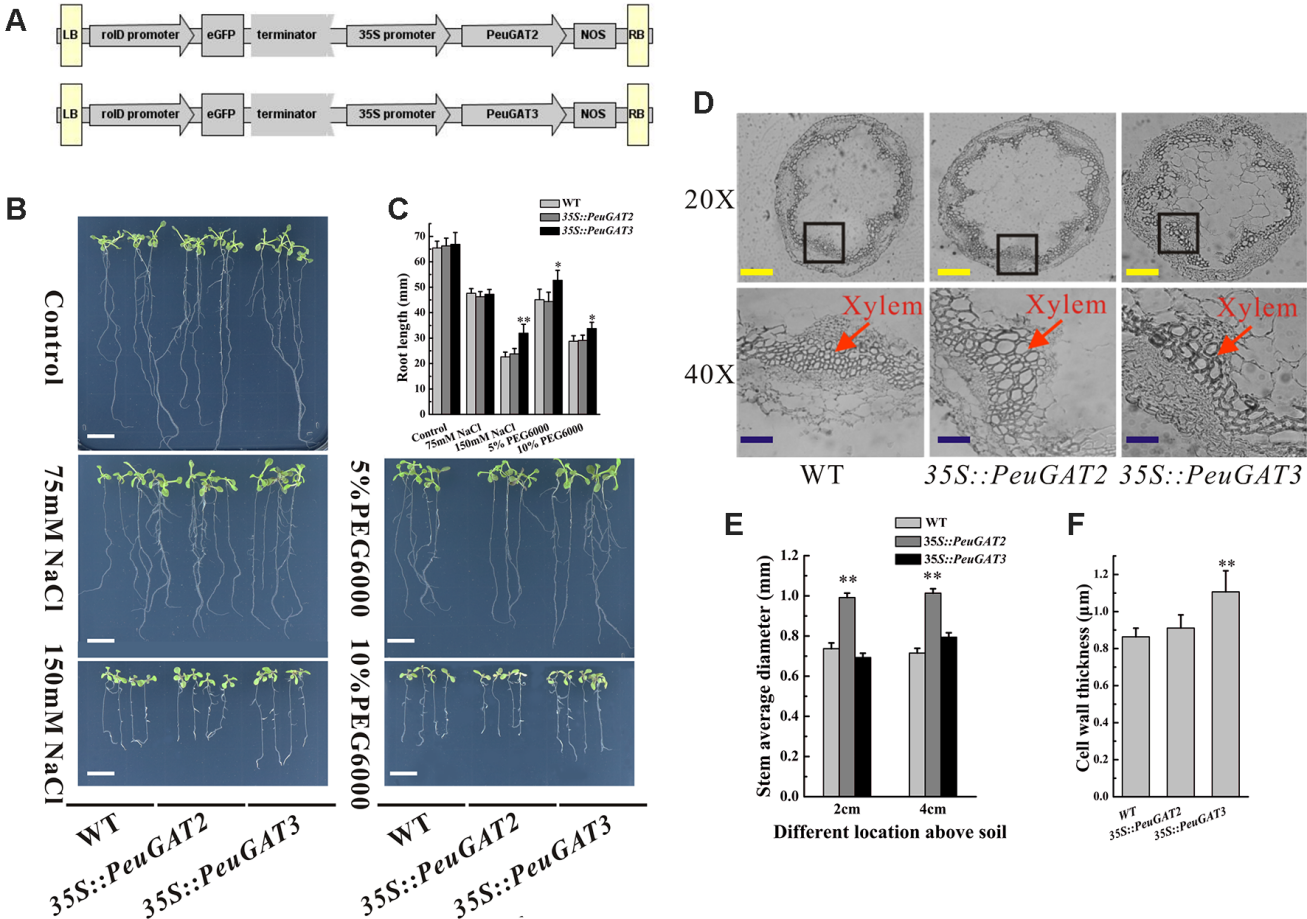
Morphology phenotype of transgenic *Arabidopsis*. **(A)** Constructs of vectors used in transgenic *Arabidopsis* and poplar. **(B)** Phenotypes of wild-type and transgenic *Arabidopsis* under salt and drought stresses. **(D)** Stem anatomical structures of wild-type and transgenic *Arabidopsis*. The root length **(C)** diameter of stem **(E)** and cell wall thickness **(F)** of wild-type and transgenic *Arabidopsis*. Each sample was analyzed in triplicate. All values are means ± SE (*n* = 20). Values labeled with different letters differ significantly (*p < 0.05, **p < 0.01). Bars: white, 10 mm; yellow, 20 µm; blue, 40 µm.

**Figure 5:**
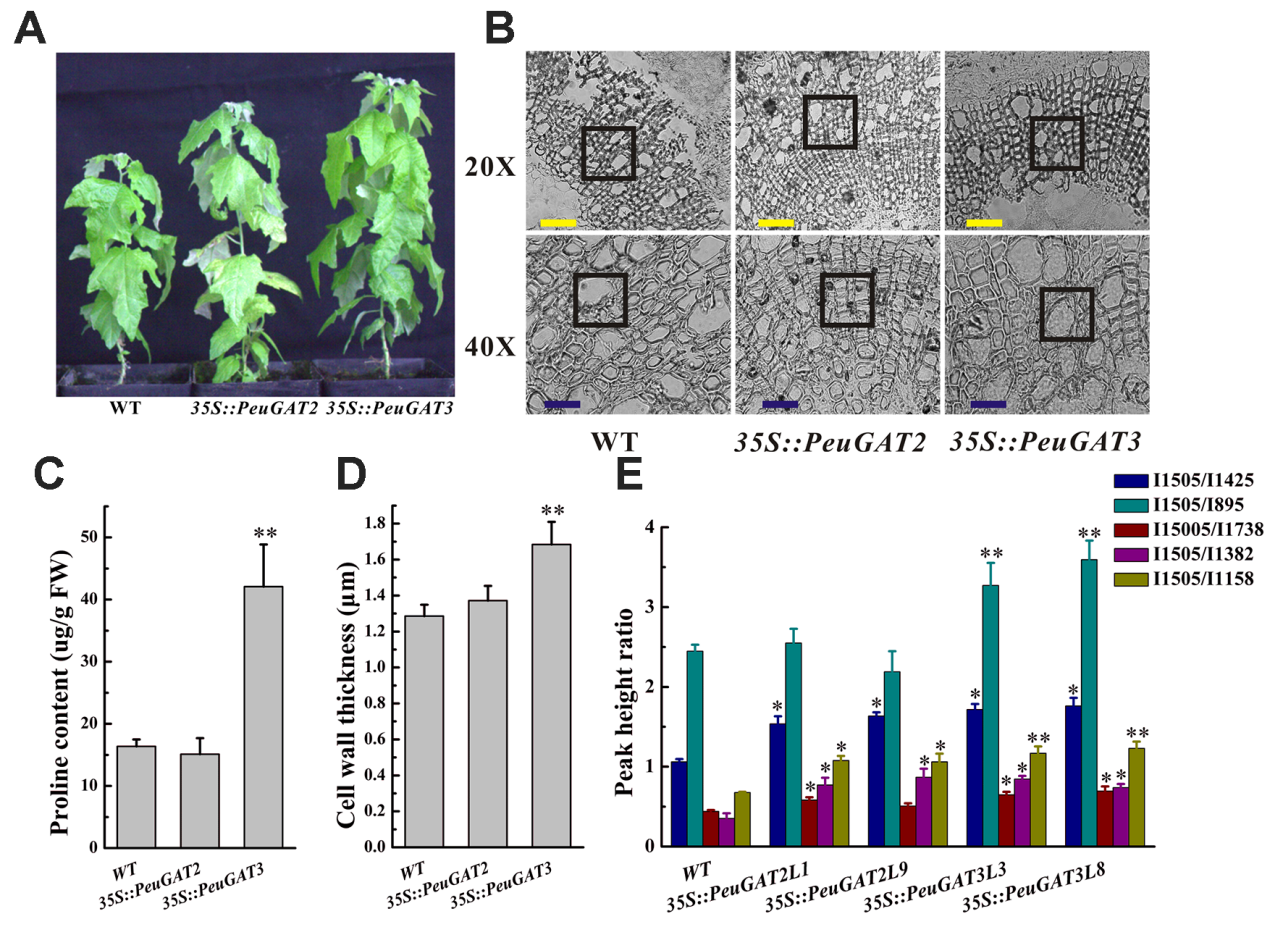
The phenotype **(A)** and stem anatomy **(B)** of wild and transgenic poplars overexpressing *PeuGAT2* and *PeuGAT3*. **(C)** Proline content in wild-type and transgenic poplar leaves. **(D)**The cell wall thickness of wild and transgenic poplars overexpressing *PeuGAT2* and *PeuGAT3*. **(E)**The wood composition of wild and transgenic poplars overexpressing *PeuGAT2* and *PeuGAT3*. I1505/I1382 and I1505/I1158 represent the ratio of lignin to total cellulose, while I1505/I1425, I1505/I895, and I1505/I1738 represent the relative content of lignin, the lignin to cellulose ratio, and the lignin to hemicellulose ratio, respectively. Values labeled with different letters differ significantly (*p < 0.05 and **p < 0.01). Bars: yellow, 20 µm; blue, 40 µm.

To investigate the differences between the function of *PeuGAT2* and *PeuGAT3* in plants, they were overexpressed in *Arabidopsis* (**Figure 4A**) and *Populus* (**Figure 5A**) species through transgenic methods. Under normal condition, the WT and two transgenic *Arabidopsis* lines showed similar phenotype. In the cases of abiotic stress treatment, except *PeuGAT3* overexpression lines, the root growths of the remaining *Arabidopsis* lines were significantly inhibited (**Figures 4B, C**). However, in the process of germination, there was no significant difference between wild type and two overexpressing Arabidopsis lines, whether in normal condition or salt stress conditions (**Figure S3**). Furthermore, in vegetative growth phase, the *35S::PeuGAT2* line exhibited significantly (*p* < 0.05) thicker stems as determined by measuring the stem diameter at heights of 2 and 4 cm above the soil (**Figure 4E**), while *35S::PeuGAT3* line exhibited a more thicker cell wall (**Figures 4D, F**). In transgenic *Populus* lines, similar phenotypes were observed but the difference were less pronounced. Lines overexpressing *PeuGAT3* produced ligneous tissues with thicker cell walls than were seen in lines overexpressing *PeuGAT2* (**Figures 5B, D**), indicating that the neo-functionalization of *PeuGAT3* may confer plasticity that allows trees to limit water losses.

To characterize the morphological and anatomical differences between the transgenic *Populus* and *Arabidopsis* lines, their wood composition was studied by FTIR (BRUKER TENSOR 27, Germany), and their anatomical structure was studied using a dissecting microscope. *35S::PeuGAT2* overexpression in poplars increased the size of xylem cells in the stem, whereas overexpression of *35S::PeuGAT3* caused the cell walls of stem xylem cells to thicken significantly (**Figures 5B, D**). Similar results were obtained in the transgenic *Arabidopsis* lines: lines overexpressing *35S::PeuGAT2* produced larger xylem cells, whereas those overexpressing *35S::PeuGAT3* produced thicker xylem cell walls (**Figures 4D, F**). In addition, five key IR spectroscopic ratios were determined to characterize the wood composition of the transgenic and WT *Populus* plants (**Figure 5E**). Measurements of the I1505/I1425 ratio (i.e., the ratio of lignin to cellulose) indicated that transgenic plants had higher lignin contents than did their WT counterparts. The I1505/I895 ratio (i.e., the ratio of lignin to hemicellulose) was significantly higher in lines overexpressing *35S::PeuGAT3* than those overexpressing *35S::PeuGAT2*, but the I1505/I1738; I1505/I1382 and I1505/I1158 ratios (representing the ratio of lignin to total cellulose) were similar for both transgenic lines.

The proline content of the leaves typically reflects plants’ tolerance for various biotic and abiotic stresses. We therefore measured the proline levels in the leaves of the WT and transgenic poplars. As shown in **Figure 5C**, the *35S::PeuGAT3* poplars had significantly higher levels of proline than WT poplars, but the *35S::PeuGAT2* poplars had similar leaf proline levels to WT poplars.

## Discussion

### The GAT genes in *Populus* Expanded in Specific Genus

The novel gene arose through gene duplication and divergence provide important genetic resources that drive evolution (Kondrashov et al., 2002). In the immediate aftermath of a duplication event, the duplicated genes will typically exhibit almost complete functional redundancy. Copies that acquire detrimental mutations are likely to be eliminated by purifying selection, but those that diverge functionally may be retained (Force et al., 1999), potentially leading to the emergence of novel and evolutionarily beneficial functions (Long and Langley, 1993; Long et al., 1999; Katju and Lynch, 2006; Nan et al., 2017). Some of these duplicated genes may be fixed by selection because they are adaptive, as may be the case if they increase stress tolerance and growth flexibility (Kondrashov et al., 2002). In AAAP family of plants, subfamilies GAT and ProT diversified recently from a common ancestral gene. The GAT gene family then expanded with specific genus in *Populus* but not in *Arabidopsis*, while in the ProT family, the opposite is true. In *Arabidopsis*, only one AtGAT gene has been identified and has a high affinity for GABA but cannot transport proline (Schwacke et al., 1999), suggesting that GAT and ProT subfamilies have diversified completely in function. Whereas in poplar, the genus-specific expansion of *GATs* would improve poplar species adaptation or contribute the formation of polymorphic traits, such as wood property and tolerance to stress. Of them, *PeuGAT3*, sister gene of *PeuGAT2*, underwent rapid evolution and have suffered from positive selection. Furthermore, no *PeuGAT3* orthologs have been found in the closely related poplars, indicating *PeuGAT3* arose lineage specific through a duplication event (Tautz and Domazet-Lošo, 2011). Therefore, *PeuGAT3* has been identified as a novel gene that created through gene duplication or divergence event. It is clear that the genus-specific expansion of GAT in *Populus* and subsequent diversification provide important genetic resource in the formation of specific adaptive traits (Shukla et al., 2014), while the divergence of novel *PeuGAT3* in protein function/structure might define its function better than in expression pattern.

### Functional Divergence Between *PeuGAT2* and *PeuGAT3* Occurred in Multiple Levels

The functional divergence among paralogous genes (including novel genes) plays an important role in plant adaptation (Lynch et al., 2001; He and Zhang, 2005). The proteins encoded by duplicated genes have diversified in their expression patterns and/or biochemical functions, leading to changes in gene structure and the properties of the corresponding proteins (Force et al., 1999; Nan et al., 2017), site-specific regulatory modification of proteins, variation of splicing sites among isoforms (Bergström et al., 2014), and protein binding specificity (Hittinger and Carroll, 2007; Gagnon-Arsenault et al., 2013). In the desert poplars species, two paralogous *PeuGAT2* and *PeuGAT3* have diversified not only in expression pattern during salt treatment but also in gene structure and function. First, *PeuGAT3* express in phloem and leaves, especially in salt treatment, while *PeuGAT2* express in lignin. The novel genes express in tissue specific and involve in response to environmental stresses and in formation of lineage-specific traits (Arendsee and Li, 2014). Second, *PeuGAT3* lacks one motif found in *PeuGAT2*, which lead to lack of a transmembrane domain in PeuGAT3. Finally, the overexpression of *PeuGAT2* improved the *Arabidopsis* rapid growth of the aerial parts by keep the size of cells in xylem, while the overexpression of *PeuGAT3* in *Arabidopsis* and poplar enhanced the accumulation of proline in leaves and lignin content in the xylem tissues, which enable cells to better regulate their osmotic potential and further accelerate proline transport (Yancey, 2005; Verslues et al., 2006). Meanwhile, the higher expression of *PeuGAT3* than *PeuGAT2* in leaves could improve ion homeostasis between leaves and roots (Foyer and Noctor, 2005). Obviously, in poplar, PeuGAT3 maybe have more affinity to proline than PeuGAT2.

### *PeuGAT3* Remains the Proline Transport That May Improve the Stress Tolerance in *P. euphratica*

Proline is a beneficial solute whose accumulation allows plant cells to increase their osmolarity and thereby avoid damage caused by osmotic stress (Yancey, 2005; Lehmann et al., 2010; Szabados and Savoure, 2010). In *Arabidopsis*, three ProTs copies involved in proline transporting are known to exhibit differential expression in different tissues under various stresses (Grallath et al., 2005). It might be expected that a proline transporter would be induced by stress when proline levels are also increasing. In *Populus*, only one ProT protein was responsible for proline transport. However, overexpression *PeuGAT3*, a closely related gene of *PeuProTs*, can increase proline content in poplar and improve tolerance to salt and drought in *Arabidopsis*. Therefore, this novel gene *PeuGAT3* would be responsible for partial proline transport, and this greatly contributes to the adaptation of *P. euphratica* to arid and high salt environments. Additionally, overexpression *PeuGAT3* increased the thickness of cell walls in the ligneous tissues. This may be an adaptive anatomical feature that helps plants such as *P. euphratica* to reduce the risk of cavitation in arid climates (Tyree and Sperry, 1989; Sperry et al., 2008).It is well-known that *P. euphratica* do not fall down and even rot when it is dead because of its hard tree trunk. *PeuGAT3* overexpression increased the lignin content in the xylem tissues of transgenic poplar lines to a greater degree than was observed for *PeuGAT2* overexpression. High lignin levels in the xylem of transgenic poplars could enhance their resistance to the erosion by harsh environment, indicating that this activity of novel gene *PeuGAT3* in *P. euphratica* may partly explain why the trunks of dead *P. euphratica* trees often persist without rotting for extended periods of time. However, the transgenic poplars examined in this work were too young to study their resistance. It would certainly be desirable to investigate this in future.

## Data Availability

All datasets for this study are included in the manuscript and the **Supplementary Files**.

## Author Contributions

DW designed the experiments and wrote the manuscript. XB, JX, XS, and ZN performed the experiments. WL and CG contributed to the data analyses.

## Conflict of Interest Statement

The authors declare that the research was conducted in the absence of any commercial or financial relationships that could be construed as a potential conflict of interest.
