# Supplementary figures and images for: A Novel Gene Coding γ-Aminobutyric Acid Transporter May Improve the Tolerance of Populus euphratica to Adverse Environments
Source: Front Plant Sci. 2019 Sep 11;10:1083. doi: 10.3389/fpls.2019.01083 (PMC6749060; doi:10.3389/fpls.2019.01083)

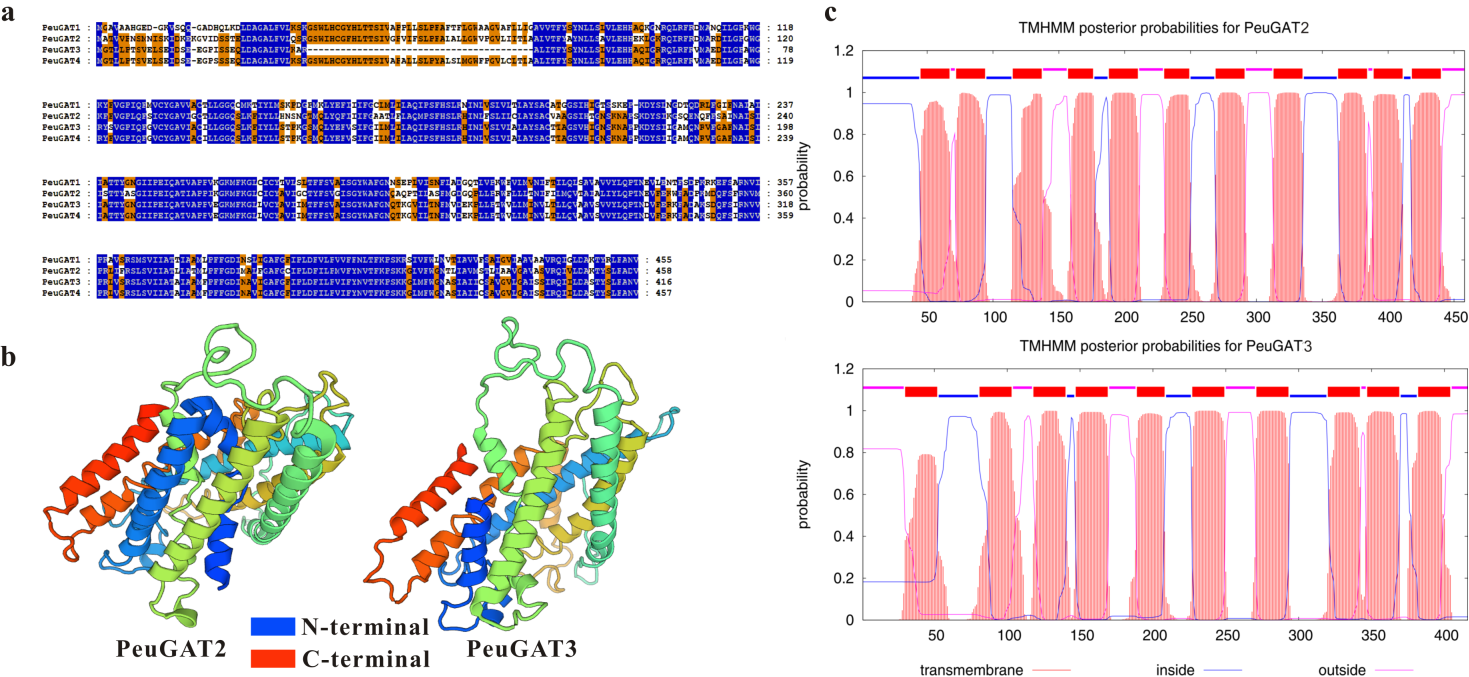

Supplement: Supplementary file 1 [file Image_1.tif]

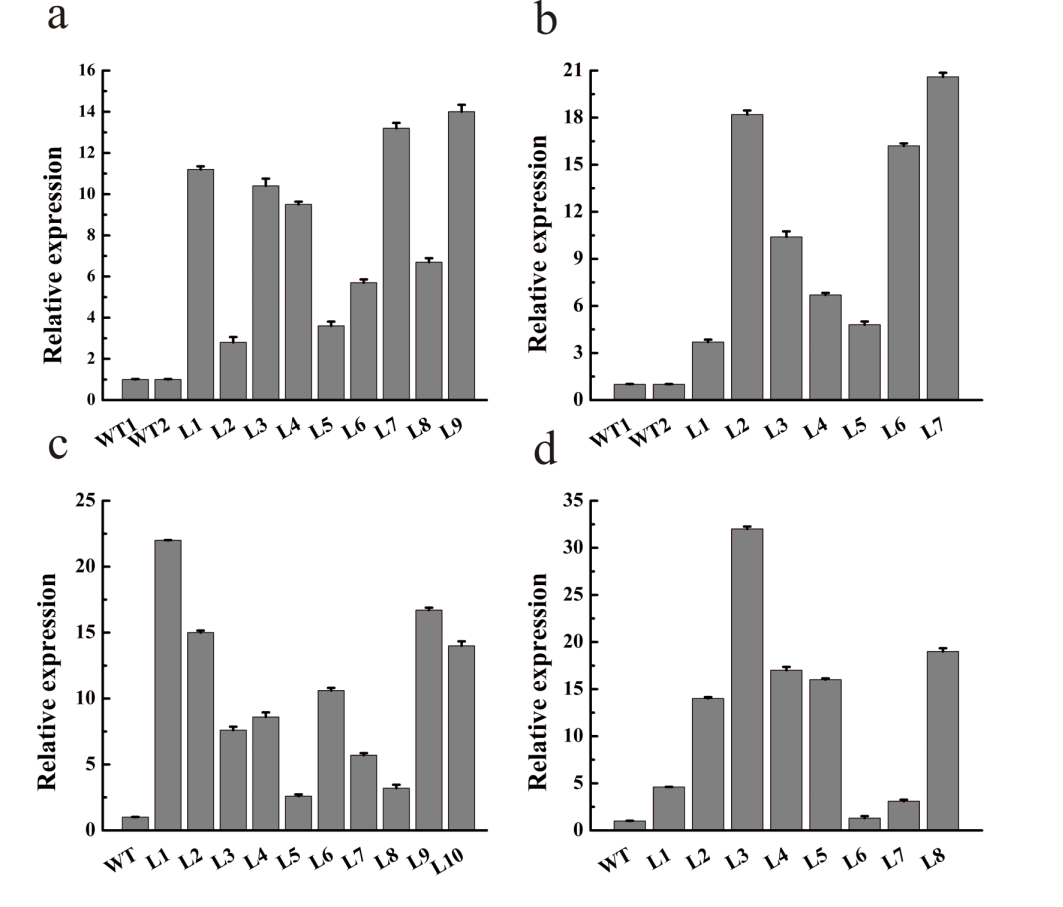

Supplement: Supplementary file 2 [file Image_2.tif]

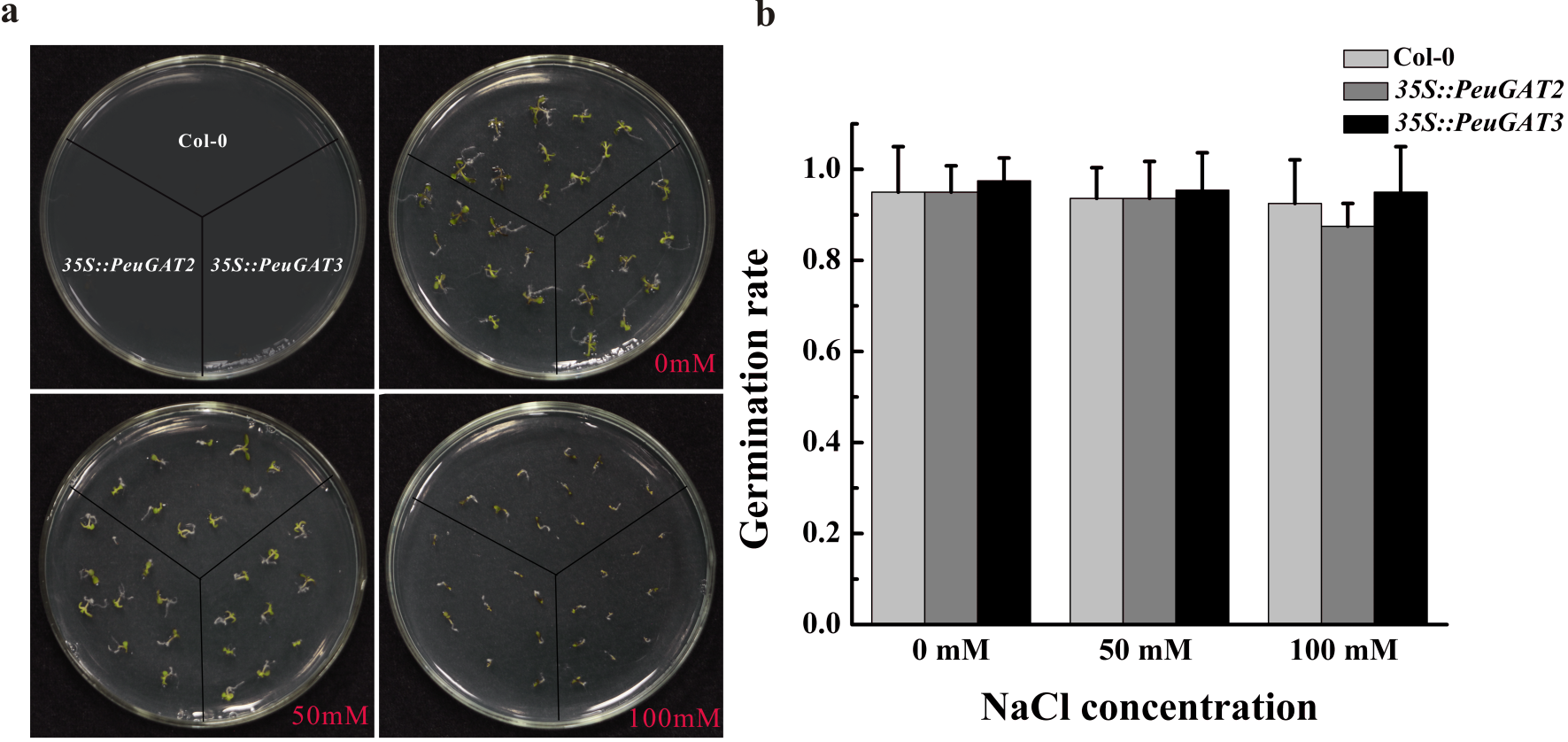

Supplement: Supplementary file 3 [file Image_3.tif]
