# Supplementary material for: A Novel Gene Coding γ-Aminobutyric Acid Transporter May Improve the Tolerance of Populus euphratica to Adverse Environments
Source: Front Plant Sci. 2019 Sep 11;10:1083. doi: 10.3389/fpls.2019.01083 (PMC6749060; doi:10.3389/fpls.2019.01083)
Supplement: Supplementary file 4 [file Table_1.docx]

Supplemental Table 1 the primers used in the study

| Primer name | Forward (5`-3`) | Reverse (5`-3`) |
| --- | --- | --- |
| PeuGAT2(for RT) | TAGCCTTCCCTTTGCTCTGG | CACAAAAAACTTGCCCCATC |
| PeuGAT3(for RT)  ACTIN(for RT) | TGGTCCGATTCAGTTTGGTG  GATTCTATGGGTGGTGGTGC | GCCGTAGGGAGTGGAAGGAT  CAGGCTGAGGTCTCGTTCG |
| PeuGAT2(pDONOR) | AAAAAAGCAGGCTTCATGCAACGGTGGTTCCAAC | CAAGAAAGCTGGGTTACGTCAGCGAACAAGCTATAAG |
| PeuGAT3(pDONOR) | AAAAAAGCAGGCTTCATGGGAACTCTACTTCCTACTTC | CAAGAAAGCTGGGTTCATTAGCAAACAAGCTATACGT |
| AttB | GGGGACAAGTTTGTACAAAAAAGCAGGCT | GGGGACCACTTTGTACAAGAAAGCTGGGT |

Supplemental Table 2. Ks-based estimation of timing of *PeuGAT1* and *PeuGAT2* duplication.

| Gene A | Gene B | Ka | Ks | Ka/Ks | Time (Mya) |
| --- | --- | --- | --- | --- | --- |
| *PeuGAT2* | *PeuGAT3* | 0.4317 | 0.3675 | 1.1747 | 49 |

Supplemental Table 3. Accession numbers of amino acid transporter gene sequences used in multiple sequence alignment and phylogenetic analysis. Protein sequences were obtained from NCBI. At, *Arabidopsis thaliana*; Ptr, *Populus trichocarpa*; Ppr, *P. pruinosa*; CCG, *P. euphratica*; willow, *Salix suchowensis*; PAYT, *P.* *alba* var. *pyramidalis.*

| Name | Genome Name | Name | Genome Name |
| --- | --- | --- | --- |
| *AtAGAT* | AT5G41800.1 | *At*ProT1 | AT2G39890.1 |
| *AtProT2* | AT3G55740.1 | *AtProT3* | AT2G36590.1 |
| *AtANT1* | AT2G42005.1 | *AtANT2* | AT3G11900.1 |
| *AtANT3* | AT4G38250.1 | *AtANT4* | AT5G15240.1 |
| *AtLHT1* | AT1G48640.1 | *AtLHT2* | AT1G24400.1 |
| *AtLHT3* | AT1G67640.1 | *AtLHT4* | AT1G71680.1 |
| *AtLHT5* | AT1G25530.1 | *AtLHT6* | AT4G35180.1 |
| *AtAAP1* | AT1G58360.1 | *AtAAP2* | AT5G09220.1 |
| *AtAAP3* | AT1G77380.1 | *AtAAP4* | AT5G63850.1 |
| *AtAAP5* | AT1G44100.1 | *AtAAP6* | AT5G49630.1 |
| *AtAAP7* | AT5G23810.1 | *AtAAP8* | AT1G10010.1 |
| \| *PtrGAT1* \| POPTR_0009G149900.1 \| \| --- \| --- \| | POPTR_0009G149900.1 | *PtrGAT2-1* | POPTR_0001G093600.1 |
| *PtrGAT2-2* | POPTR_0003G138100.1 | *PtrAGAT3* | POPTR_0008G026600.1 |
| *PtrGAT4* | POPTR_0008G026700.1 | *PtrProT1* | POPTR_0005G219300.1 |
| *PtrANT1* | POPTR_0008G036300.1 | *PtrANT2* | POPTR_0010G226000.1 |
| *PtrANT3* | POPTR_0016G100300.1 | *PtrLHT1* | POPTR_0002G012900.1 |
| *PtrLHT2* | POPTR_0004G181000.1 | *PtrLHT3* | POPTR_0004G181200.1 |
| Name | Genome Name | Name | Genome Name |
| *PtrLHT4* | POPTR_0009G140800.1 | *PtrLHT5* | POPTR_0014G036500.1 |
| *PtrAAP1* | POPTR_0002G079400.1 | *PtrAAP2* | POPTR_0002G079500.1 |
| *PtrAAP3* | POPTR_0009G133600.1 | *PtrAAP4* | POPTR_0007G100100.1 |
| *PtrAAP5* | POPTR_0002G079700.1 | *PtrAAP6* | POPTR_0002G112100.1 |
| *PtrAAP7* | POPTR_0001G470000.1 | *PtrAAP8* | POPTR_0006G236000.1 |
| *PtrAAP9* | POPTR_0005G181500.1 | *PtrAAP10* | POPTR_0005G181600.1 |
| *PtrAAP11* | POPTR_0005G068900.1 | *PtrAAP12* | POPTR_0009G085000.1 |
| *PtrAAP13* | POPTR_0011G167300.1 | *PtrAUX1* | POPTR_0008G066400.1 |
| *PtrAUX2* | POPTR_0016G113600.1 | *PtrAUX3* | POPTR_0002G087000.1 |
| *PtrAUX4* | POPTR_0005G174000.1 | *PtrAUX5* | POPTR_0004G172800.1 |
| *PtrAUX6* | POPTR_0006G098300.1 | *PtrAUX7* | POPTR_0009G132100.1 |
| *PtrAUX8* | POPTR_0010G191000.1 | *PeuGAT1* | CCG017051.1 |
| *PeuGAT2* | CCG029175.1 | *PeuGAT3* | CCG029176.1 |
| *PeuGAT4* | CCG029178.1 | *PeuProT1* | CCG006477.1 |
| *PeuANT1* | CCG000347.1 | *PeuANT2* | CCG004159.1 |
| *PeuANT3* | CCG004161.1 | *PeuANT4* | CCG004416.1 |
| *PeuANT5* | CCG016651.1 | *PeuANT6* | CCG028476.1 |
| *PeuLHT1* | CCG004935.1 | *PeuLHT2* | CCG004936.1 |
| *PeuLHT3* | CCG004937.1 | *PeuLHT4* | CCG010321.1 |
| *PeuLHT5* | CCG017126.1 | *PeuLHT6* | CCG029326.1 |
| *PeuAAP1* | CCG012180.1 | *PeuAAP2* | CCG012181.1 |
| *PeuAAP3* | CCG017187.1 | *PeuAAP4* | CCG017591.1 |
| *PeuAAP5* | CCG020055.1 | *PeuAAP6* | CCG023373.1 |
| *PeuAAP7* | CCG023374.1 | *PeuAAP8* | CCG026117.1 |
| *PeuAAP9* | CCG033471.1 | *PeuAAP10* | CCG033476.1 |
| *PeuAUX1* | CCG000186.1 | *PeuAUX2* | CCG004499.1 |
| *PeuAUX3* | CCG020463.1 | *PeuAUX4* | CCG023963.1 |
| *PeuAUX5* | CCG026200.1 | *PeuAUX6* | CCG027310.1 |
| *PprGAT1* | PPR003069.1 | *PprGAT2-1* | PPR003897.1 |
| *PprGAT2-2* | PPR013180.1 | *PprGAT3* | PPR003068.1 |
| *PprProT1* | PPR034533.1 | *PprANT1* | PPR003796.1 |
| *PprANT2* | PPR006402.1 | *PprANT3* | PPR008890.1 |
| *PprANT4* | PPR011926.1 | *PprANT5* | PPR018531.1 |
| *PprANT6* | PPR030836.1 | *PprANT7* | PPR031228.1 |
| *PprANT8* | PPR031829.1 | *PprLHT1* | PPR014176.1 |
| *PprLHT2* | PPR029782.1 | *PprLHT3* | PPR029784.1 |
| *PprAAP1* | PPR001550.1 | *PprAAP2* | PPR003041.1 |
| *PprAAP3* | PPR004765.1 | *PprAAP4* | PPR004767.1 |
| *PprAAP5* | PPR007534.1 | *PprAAP6* | PPR015283.1 |
| *PprAAP7* | PPR016712.1 | *PprAAP8* | PPR023175.1 |
| *PprAUX1* | PPR016560.1 | *PprAUX2* | PPR016851.1 |

| Name | Genome Name | Name | Genome Name |
| --- | --- | --- | --- |
| *PprAUX3* | PPR017474.1 | PprAUX4 | PPR018698.1 |
| *PprAUX5* | PPR019306.1 | PprAUX6 | PPR019737.1 |
| *PprAUX7* | PPR023473.1 | *PalGAT1* | PAYT011216.1 |
| *PalGAT2-1* | PAYT038149.1 | *PalGAT2-2* | PAYT029375.1 |
| *PalGAT3* | PAYT010518.1 | *PalGAT4* | PAYT010517.1 |
| *PalProT1* | PAYT009119.1 | *PalANT1* | PAYT005580.1 |
| *PalANT2* | PAYT010628.1 | *PalANT3* | PAYT016636.1 |
| *PalANT4* | PAYT025579.1 | *PalANT5* | PAYT028881.1 |
| *PalLHT1* | PAYT007610.1 | *PalLHT2* | PAYT007611.1 |
| *PalLHT3* | PAYT007624.1 | *PalLHT4* | PAYT011313.1 |
| *PalLHT5* | PAYT012657.1 | *PalLHT6* | PAYT016251.1 |
| *PalAAP1* | PAYT002985.1 | *PalAAP2* | PAYT002987.1 |
| *PalAAP3* | PAYT002989.1 | *PalAAP4* | PAYT011390.1 |
| *PalAAP5* | PAYT013975.1 | *PalAAP6* | PAYT014025.1 |
| *PalAAP7* | PAYT015422.1 | *PalAAP8* | PAYT018197.1 |
| *PalAAP9* | PAYT020507.1 | *PalAAP10* | PAYT023787.1 |
| *PalAAP11* | PAYT026810.1 | *PalAAP12* | PAYT029215.1 |
| *PalAAP13* | PAYT029248.1 | *PalAAP14* | PAYT029259.1 |
| *PalAAP15* | PAYT032964.1 | *PalAUX1* | PAYT001171.1 |
| *PalAUX2* | PAYT008556.1 | *PalAUX3* | PAYT011410.1 |
| *PalAUX4* | PAYT022973.1 | *PalAUX5* | PAYT027638.1 |
| *PalAUX6* | PAYT027879.1 | *WiGAT1* | willow_GLEAN_10023923 |
| *WiGAT2-1* | willow_GLEAN_10023689 | *WiGAT2-2* | willow_GLEAN_10025381 |
| *WiGAT3* | willow_GLEAN_10024648 | *WiGAT4* | willow_GLEAN_10024649 |
| *WiProT1* | willow_GLEAN_10019411 | *WiANT1* | willow_GLEAN_10011976 |
| *WiANT2* | willow_GLEAN_10015150 | *WiANT3* | willow_GLEAN_10019641 |
| *WiANT4* | willow_GLEAN_10024709 | *WiLHT1* | willow_GLEAN_10015351 |
| *WiLHT2* | willow_GLEAN_10015354 | *WiLHT3* | willow_GLEAN_10016764 |
| *WiLHT4* | willow_GLEAN_10022610 | *WiAAP1* | willow_GLEAN_10002023 |
| *WiAAP2* | willow_GLEAN_10002326 | *WiAAP3* | willow_GLEAN_10003787 |
| *WiAAP4* | willow_GLEAN_10013919 | *WiAAP5* | willow_GLEAN_10020803 |
| *WiAAP6* | willow_GLEAN_10020804 | *WiAAP7* | willow_GLEAN_10021045 |
| *WiAAP8* | willow_GLEAN_10024035 | *WiAAP9* | willow_GLEAN_10026258 |
| *WiAUX1* | willow_GLEAN_10012026 | *WiAUX2* | willow_GLEAN_10018256 |
| *WiAUX3* | willow_GLEAN_10019387 | *WiAUX4* | willow_GLEAN_10024047 |
| *WiAUX5* | willow_GLEAN_10024930 | *WiAUX6* | willow_GLEAN_10026642 |
